# Hemocoagulative Modifications after Laparoscopic Surgery at Different Pneumoperitoneum Pressure Settings

**DOI:** 10.29337/ijsp.173

**Published:** 2022-06-14

**Authors:** Intagliata Eva, Vecchio Rosario, Saitta Cesare, Vizzini Clarissa, Lo Presti Federica, Cacciola Rossella Rosaria, Cacciola Emma, Vecchio Veronica

**Affiliations:** 1 Department of General Surgery and Medical Surgical Specialties, University of Catania, Policlinico “G. Rodolico – San Marco”, Via S. Sofia 78, 95123 Catania, Italy; 2 Department of Biomedical Sciences, Humanitas University, Via Rita Levi Montalcini 4, 20090 Pieve Emanuele, Milan, Italy; 3 Department of Biomedical Science, Hematologic Unit, University of Catania, Policlinico “G. Rodolico – San Marco”, Via S. Sofia 78, 95123 Catania, Italy; 4 Department of Medical Sciences, Surgical Sciences and Advanced Technologies, Hemostasis Unit, University of Catania, Italy, Policlinico “G. Rodolico – San Marco”, Via S. Sofia 78, 95123 Catania, Italy

**Keywords:** pneumoperitoneum, pneumoperitoneum pressure, laparoscopy, hemocoagulation

## Abstract

**Background:**

Many of the effects of pneumoperitoneum on cardiovascular, respiratory and metabolic systems have been discussed in Literature, but very little is known about the variations of the hemocoagulative parameters in patients undergoing laparoscopic surgery. The purpose of this study is to analyze the variations of the hemocoagulative parameters in patients undergoing elective laparoscopic cholecystectomy for symptomatic gallbladder stones. An eventual statistically significant difference linked to different pressure settings of pneumoperitoneum will allow selecting a specific intrabdominal pressure for a more adequate treatment with a lower incidence of pneumoperitoneum related complications.

**Materials and Methods:**

The clinical trial was conducted on 43 patients assigned in two groups based on the intra-abdominal pressure: group A, 27 patients, 12 mmHg, and group B, 16 patients, 8 mmHg. Hemoglobin, hematocrit, platelets count, PT ratio, aPTT, Fibrinogen, D-dimer, Von Willebrand factor, Factor II, Lupus Anticoagulant, Antithrombin III, Protein C, Protein S, Anticardiolipin IgG and IgM, anti-beta 2-Glicoprotein IgG and IgM were evaluated.

**Results:**

For group A, patient’s variations were observed for D-dimer, Factor II, von Willembrand factor and protein C reactive, while for patients belonging to group B the parameters most affected were PT ratio, anti-thrombin III and protein C reactive.

D-dimer values increased significantly in group A, a statistically significant decrease in anti-thrombin III levels was detected in group B, and a statistically significant difference in PT ratio in patients belonging to group B was observed.

**Conclusion:**

The statistical analysis showed no significant difference in the post-operative parameters when comparing the two groups of patients. Alterations of the coagulation parameters were present between pre- and post-operative data within the same group, namely a higher abdominal pressure is linked to a prothrombotic state. The question is worthy of further studies.

**Highlights:**

- Variations of the hemocoagulative parameters in patients undergoing laparoscopy are still a matter of study;
- Pneumoperitoneum seems to cause alterations in the hemocoagulative parameters, which could be influenced by the pneumoperitoneum pressure;
- An eventual statistically significant difference linked to different pressure settings of pneumoperitoneum will allow selecting a specific intrabdominal pressure for a more adequate surgical treatment with a lower incidence of pneumoperitoneum related complications.

## 1. Introduction

A fundamental step of the laparoscopic surgery is the insufflation of dioxide carbon inside the peritoneal cavity to distend the abdominal wall in order to create enough space for surgeons’ maneuver.

Many of the effects of pneumoperitoneum on cardiovascular, respiratory and metabolic systems have been discussed in Literature.

The purpose of this study is to analyze the hemocoagulative parameters variations in patients undergoing laparoscopic cholecystectomy for symptomatic gallbladder stones, in order to highlight the presence or absence of a statistically significant advantage linked to different pressure settings of pneumoperitoneum. If such a correlation will be present, it would give the possibility to select a specific intrabdominal pressure, in order to offer a more adequate treatment with a lower incidence of pneumoperitoneum related complications.

## 2. Materials and Methods

The clinical trial was conducted between July 2019 and February 2020, after approval of the ethics committee of the Policlinico University Hospital of Catania, Italy.

43 patients have undergone elective laparoscopic surgery for symptomatic gallbladder stones. 22 of them have been selected retrospectively. The patients were randomized and assigned into two different groups based on the intra-abdominal pressure, specifically group A, where the laparoscopic cholecystectomy procedures were performed at an intra-abdominal pressure of 12 mmHg, and group B where the laparoscopic cholecystectomy procedures were performed at an intraabdominal pressure of 8 mmHg.

Exclusion criteria were: active immunodeficiency, chronic use of systemic corticosteroids, uncompensated diabetes mellitus, comorbidities with life expectancy less than 30 days, significant anemia, pathologies of the coagulation spectrum, presence of ascites, patients with chronic pain syndrome, need of chronic pain relief treatment, severe comorbidities (atrial fibrillation, pulmonary hypertension).

Parameters related to blood coagulation, obtained from a venous blood sample taken before anesthesiologic procedures and on the first post-operative day, were: hemoglobin (HB), hematocrit (HCT), platelets count, PT ratio, aPTT, Fibrinogen, D-dimer, Von Willebrand factor (vWF), Factor II, Lupus Anticoagulant (LAC), Antithrombin III (ATIII), Protein C (PC), Protein S (PS), Anticardiolipin (ACA) IgG and IgM, anti-beta 2-Glicoprotein IgG and IgM.

The same surgical team of our Department of Laparoscopic Surgery operated all patients. The surgical technique used was the same in all cases. All patients were treated with the same antibiotic prophylaxis, namely 3rd generation cephalosporin.

The team of anesthesiologists used the same anesthesiologic procedure for all patients, and the standard used to induce anesthesia was opioid-free total intravenous anesthesia. Muscle relaxation was induced by Rocuronium. At the end of the operation, the analgesic protocol was the same for all patients, namely paracetamol 1000 mg 3 times a day.

The data were analyzed using the IBM SPSS-package software. Different statistical tests were used depending on the distribution of the variable considered, distinguishing between normal and not normal. The normality of data distributions was checked with the Kolmorov-Smirnov test.

The descriptive part of the analysis was carried out by computing absolute frequencies and percentages for the qualitative variables. For the quantitative analysis, mean and standard deviation were computed if the variable under consideration was normally distributed, while median and interquartile range (IQR) were used for variables not normally distributed.

For the comparison between the pre- and post-surgery distributions of the values of the parameters, the t-Student paired-test was used for normally distributed data, while the Wilcoxon signed-rank test was used for non-normal data.

For the comparison between the two groups, the X2-test with Yates correction or the exact Fisher test were adopted for qualitative variables.

For quantitative variables instead, the t-Student test for unpaired data and the U-test of Mann-Whitney were used respectively depending on whether the variable followed or not a normal distribution. Differences leading to a p-value ≤ 0.05 were considered significant.

## 3. Results

A total of 43 patients were divided into group A and group B, consisting respectively of 27 and 16 patients each. 26 patients (60.5%) were female and 17 (39.5%) were male. The patients’ age ranged between 15 and 85 years, with an average age of 51 years. The average operating time was 96 minutes.

The pre-operative values of the parameters considered, had similar distributions for patients belonging to the two groups A and B, with the exception of ATIII whose compatibility was marginal (p = 0.019), thus demonstrating the absence of bias. Therefore, the two groups were homogeneous.

Data from the two groups of patients were first analyzed separately, searching for variations between the pre- and post-operation data within each group.

Tables 1 and 2 summarize the pre- and post-operative data of the two groups, and Table 3 reports the comparison between the two groups in the post-operative period.

**Table 1:** Group A data.


	PRE-SURGERY			POST-SURGERY			P
	
	N	MEAN/MEDIAN	σ/IQR	N	MEAN/MEDIAN	σ/IQR	

HB	27	12,8	2,2	27	11,8	1,7	0,005

HTC	27	37,1	6,3	27	34,2	33,6–38,40	NS

PLATELETS	27	247,2	80,9	27	246,3	79,6	NS

PT ratio	27	1,2	1,100–1,300	27	1,2	1,1–1,3	NS

APTT	27	29	26–33	26	29,5	26,5–31	NS

FIBRINOGEN	27	317,3	78,5	25	309	261–352	NS

D-DIMER	24	168	91,5–248,75	23	330	236–730	0,004

FACTOR II	12	120	47	10	86,2	14,1	0,046

vWF	12	163,4	44,8	9	204,3	117,9–213,35	0,021

ATIII	25	90,6	15	22	86,4	12,9	NS

PC	24	95,8	20,5	22	90,1	21,3	NS

PS	24	62,9	29,2	22	68,5	19,9	NS

LAC	24	1,2	0,2	21	1,2	0,2	NS

ACA IgG	21	0,4	0–0,85	19	0,5	0–1	NS

ACA IgM	20	2,3	0,675–4,125	19	2,5	0,7–4,4	NS

ANTI β2 IgG	21	0,5	0–1,600	18	0,3	0–0,97	NS

ANTI β2IgM	20	1,1	0,225–2,400	18	1,6	0,37–3,2	NS

PCR	13	4,5	2,400–11,755	23	15,7	10,87–32,5	0,012


**Table 2:** Group B data.


	PRE-SURGERY			POST-SURGERY			P
	
	N	MEAN/MEDIAN	σ/IQR	N	MEAN/MEDIAN	σ/IQR	

HB	16	12,6	1,7	16	11,4	1,4	0,001

HTC	16	38,1	4,9	16	35,2	3,9	0,001

PLATELETS	16	260,5	233,5–340,5	16	268	192,25–318,75	NS

PT ratio	16	1,1	1,07–1,14	16	1,2	0,1	0,023

APTT	16	32,5	26,75–34	16	29,5	28–32	NS

FIBRINOGEN	16	325,9	104,7	16	341,6	109,1	NS

D-DIMER	12	213	157–747	13	419,2	201,9	NS

FACTOR II	6	91,7	18	8	87,2	13,9	NS

vWF	6	171,8	51	8	183,5	48,7	NS

ATIII	12	101,9	9,4	14	94,5	14,1	0,01

PC	12	109	28,5	13	96,5	20,5	NS

PS	12	80,2	14,5	12	73,6	17,7	NS

LAC	12	1,2	0,2	12	1,2	1,07–1,29	NS

ACA IgG	11	0,3	0–1	12	1,3	0,12–4,375	NS

ACA IgM	11	0,6	0,100–2,400	12	1,9	0,32–9,8	NS

ANTI β2 IgG	11	0	0–0,800	12	0,9	0,12–0,97	NS

ANTI β2IgM	11	0,7	0,100–1,400	12	1,2	0,57–3,77	NS

PCR	7	2,4	1,2–3,17	11	24,1	12,10–34,62	0,018


**Table 3:** Comparison between the two groups in the post-operative period.


	PRE-SURGERY			POST-SURGERY			P
	
	N	MEAN/MEDIAN	σ/IQR	N	MEAN/MEDIAN	σ/IQR	

HB	27	11,8	1,7	16	11,4	1,4	NS

HTC	27	34,2	33,6–38,4	16	35,2	3,9	NS

PLATELETS	27	246,3	79,6	16	268	192,25–318,75	NS

PT ratio	27	1,2	1,1–1,3	16	1,2	0,1	NS

APTT	26	29,5	26,5–31	16	29,5	28–32	NS

FIBRINOGEN	25	309	261–352	16	341,6	109,1	NS

D-DIMER	23	330	236–730	13	419,2	201,9	NS

FACTOR II	10	86,2	14,1	8	87,2	13,9	NS

vWF	9	204,3	117,9–213,35	8	183,5	48,7	NS

ATIII	22	86,4	12,9	14	94,5	14,1	NS

PC	22	90,1	21,3	13	96,5	20,5	NS

PS	22	68,5	19,9	12	73,6	17,7	NS

LAC	21	1,2	0,2	12	1,2	1,07–1,29	NS

ACA IgG	19	0,5	0–1	12	1,3	0,12–4,375	NS

ACA IgM	19	2,5	0,7–4,4	12	1,9	0,32–9,8	NS

ANTI β2 IgG	18	0,3	0–0,97	12	0,9	0,12–0,97	NS

ANTI β2IgM	18	1,6	0,37–3,2	12	1,2	0,57–3,77	NS

VAS	23	5,7	2,6	11	4,1	2,5	NS

PCR	14	15,7	10,87–32,5	11	24,1	12,10–34,62	NS


In group A, post-operative variations were observed for D-dimer, Factor II, vWF and PCR, while for patients belonging to group B the most affected parameters were PT ratio, ATIII and PCR.

D-dimer values increased significantly in group A (p ≤ 0.05), probably as an expression of fibrinolytic process increase. In spite of this evidence, no episodes of deep vein thrombosis or pulmonary embolism were recorded. In group B, there was a modest increase of the D-dimer without reaching the statistical significance observed in group A, probably because a lower pneumoperitoneum pressure had a lower effect on the coagulation parameters [1]. A statistically significant decrease in ATIII levels was detected in group B (p ≤ 0.05). It was interesting to observe that in the examined cases, we recorded a statistically significant difference in PT ratio in patients belonging to group B (p ≤ 0.05). While D-dimer increase is an expression of a thrombotic state, the increase in PT could be interpreted as a pro-hemorragic state.

Finally, the statistical analysis of our post-operative data showed no statistically significant difference in the parameters considered when comparing the two groups of patients. However, laparoscopy induced significant alterations of the most relevant coagulation parameters, when a comparison was made between the pre- and post-operative data within the same group.

## 4. Discussion

A fundamental step of the laparoscopic surgery is represented by the insufflation of a gas (carbon dioxide, CO_2_) inside the peritoneal cavity to allow the distention of the abdominal wall, which creates enough space for maneuver.

The pneumoperitoneum induces multiple alterations to the physiological processes due to the increase in intra-abdominal pressure and to the direct effects of CO2 at the systemic level because of its absorption. Many of the effects of pneumoperitoneum on cardiovascular, respiratory and metabolic systems have been discussed in Literature [2,3,4,5].

Laparoscopic cholecystectomy represents the gold standard for the treatment of symptomatic gallbladder stones. There is no pressure standard for inducing pneumoperitoneum. The effects of gas and the pathophysiological changes induced by CO2 have prompted surgeons to try to use low pressure to avoid the onset of some complications such as venous thromboembolism. The usual pressure range of the pneumoperitoneum is between 12 and 14 mmHg. However, international guidelines do not recommend operating at lower pressure value with the purpose of avoiding the potentially negative effects of pneumoperitoneum on heart and lungs, because either there is no clear evidence of safety or low pressure could reduce the formation of a safe working chamber [6,7].

There are many studies in the Literature concerning the effects of laparoscopy on coagulation alterations and on the lower thromboembolic risk than open surgery. Thrombotic risks during laparoscopic surgery are mostly pneumoperitoneum related. The increase in intra-abdominal pressure during pneumoperitoneum causes mechanical compression on the inferior vena cava with a reduction in venous return; moreover, the reverse Trendelenburg can induce blood stagnation in the lower limbs [5]. In addition, other Authors have recorded a state of hypercoagulability after the discharge of the patient from the hospital [8]. For this reason, some Authors recommended a thromboembolic prophylaxis for patients undergoing laparoscopy [9,10].

A pro-thrombotic effect seems not to be related exclusively to the mechanical stress of pneumoperitoneum, but also to the action of CO2 itself. It has been hypothesized that the effect of pneumoperitoneum may alter the sub-endothelial vessels wall causing changes that activate coagulation. This damage would determine an increase in endothelin synthesis that contributes to vasospasm and deep vein thrombosis. The increase in intra-abdominal pressure can also alter the liver circulation by inducing a reduction in the synthesis of vitamin K [11].

Many Authors have tried to evaluate the hemocoagulative parameters at different pressure settings of pneumoperitoneum, in order to establish the potential advantages of a lower pneumoperitoneum pressure.

Amin B et al. studied PT, aPTT, D-dimer, thrombin time and fibrinogen in patients who underwent laparoscopic surgery at a pressure of 13 mmHg, concluding that laparoscopy induces a thrombotic risk that reaches the maximum within 8 hours after the surgical procedure [12].

Sen et al. carried out a randomized study on rats divided into 4 different groups, respectively group A exposed to 6 mmHg of pressure for 1 hour, group B exposed to 6 mmHg of pressure for 2 h, group C exposed to 12 mmHg of pressure for 1 hour, group D exposed to 12 mmHg of pressure for 2 h. The study showed evidence of hyperactivation of coagulation in the groups subjected to high pressure and for a longer time, demonstrated by an increase in its factors and in fibrinogen, and a reduced activity of the fibrinolytic system [13].

Topal et al. performed a randomized study on 60 patients undergoing laparoscopic cholecystectomy divided into three different groups based on the pneumoperitoneum pressure applied, respectively of 10 –13 –16 mmHg. All patients with abnormal coagulation profile in terms of PT and aPTT were preliminarily excluded from the trial. PT, aPTT, INR and the thromboelastogram were measured 1 hour before induction of anesthesia and on the first post-operative day. The Authors found that patients belonging to the group exposed to 16 mmHg pneumoperitoneum pressure showed an alteration of the thromboelastogram in sense of increased thrombotic activity. This is the reason why a low pneumoperitoneum pressure could represent a particularly advantageous indication in patients who have additional thrombotic risk factors. Based on the results obtained, the Authors claimed that a longer exposure to a higher pneumoperitoneum pressure induced greater endothelial damage and favored the venous stasis [14].

Ido et al. showed how the flow rate at the femoral level significantly varied in function of two different settings of pneumoperitoneum pressure [15].

Donmez et al. carried out a study on randomized patients undergoing laparoscopic cholecystectomy divided into two different pressure values groups, respectively of 10 mmHg and 14 mmHg. The exclusion criteria included risk factors for deep vein thrombosis and a history of pulmonary embolism. PT, aPTT, TT, INR, fibrinogen and D-dimer were evaluated on the operative day, comparing the values measured 1 hour and 24 hours after surgery. They found a statistically significant difference between the two groups only for D-dimer in the postoperative period. The values of some of the blood-coagulation parameters were statistically different in both groups of patients when the comparison was made between the pre- and the post-operative period, highlighting that low-pressure pneumoperitoneum induced less negative effects on coagulation. For this reason, the Authors recommended to use a lower pneumoperitoneum pressure, whenever possible [1].

Similar conclusions were reached by Sharma et al. who studied the effects of pneumoperitoneum at two different pressure settings groups of 8 and 14 mmHg respectively. The values of PT, PTI (prothrombin index), INR and aPTT before and after surgical procedure were statistically different in the post-operative period in patients of both pressure groups (p = 0.007) [16].

Some Authors emphasized the compression of the inferior vena cava and the microvascular alteration induced by barotrauma as a cause of increased thrombotic risk, which reached the maximum expression within 8 hours [12,13,14].

Ido et al. instead attributed the venous stasis to the thromboembolic risk assessed by the reduced blood flow velocity in the femoral vein due to pneumoperitoneum [15].

High level of D-dimer is suggestive of venous thromboembolism [17]. In our study, we observed that the D-dimer values increased significantly in group A patients, probably as an expression of fibrinolytic process increase. In spite of this evidence, no episodes of deep vein thrombosis or pulmonary embolism were recorded. In group B, there was a modest increase of the D-dimer without reaching the statistical significance observed in group A, probably because a lower pneumoperitoneum pressure has a lower effects on the coagulation parameters [1]. A statistically significant decrease in ATIII levels was detected in group B (8 mmHg), as also reported by Sen et al. [13].

While D-dimer increase is an expression of a thrombotic state, the increase in PT could be interpreted as a pro-hemorragic state. It is indeed interesting to observe that in the examined cases, we recorded a statistically significant difference in PT ratio in patients belonging to group B (p ≤ 0.05).

These data are consistent with those reported by Donmez et al., highlighting the evidence that an increase in PT may be related to a decrease in the risk of deep vein thrombosis in patients undergoing laparoscopy [1].

Anti-phospholipid antibodies play an important role in the alteration of hemostatic process. At present, Vecchio et al. investigated the relationship between phospholipid antibodies and coagulation alterations in patients who underwent laparoscopic surgery. In their study, the patients were divided into two groups: 21 operated laparoscopically and 26 by open surgery. The pneumoperitoneum pressure chosen by the team was 12 mmHg. Blood samples were collected in the day of the surgical procedure and 24 hours after surgery for evaluation of LAC, anticardiolipin antibodies, anti-beta 2 glycoprotein, IgG and IgM. Only postoperative LAC levels showed a statically significant difference between the two groups, with higher levels recorded in the open group. The Authors believed that the increase in the antibody concentration was related to the extent of the trauma with subsequent inflammation; in fact, the laparoscopic group showed no increase in anti-phospholipid antibodies. Furthermore, it is not clear why only the LAC showed a significant difference, while this is not the case for the other antibodies. The Authors concluded that laparoscopic surgery induced less pronounced inflammation than the open technique, and that LAC could be a marker of thromboembolic risk [9,18].

Laparoscopy itself can induce alterations on the coagulation profile. The stress induced by both surgical trauma and barotrauma determined an activation of the coagulation cascade and inflammatory processes. Data in Tables 1 and 2 show that post-operative PCR values were higher than before surgery in both groups of patients, while no statistically significant difference was observed when the parameters were compared between the two groups in the postoperative phase alone (Table 3). Admittedly, it is not possible to attribute this increase to pneumoperitoneum alone, as any surgical trauma would also induce an increase in PCR. For this reason, we will aim to highlight every differences due to different pneumoperitoneum pressure settings in a larger sample. Furthermore, it was recorded an increase of vWF in group A, which expressed the interconnection between hemostasis and inflammation, being a protein of the acute phase [19].

## 5. Conclusion

The statistical analysis of our post-operative data showed no statistically significant difference in the parameters considered when comparing the two groups of patients. However, laparoscopy induced statistically significant alterations of the coagulation parameters, when a comparison was made between the pre- and post-operative data within the same group.

Although it is impossible to draw definite conclusions due to the limited sample size, the increase in these values reflects the trend of the inflammatory process induced by either pneumoperitoneum or surgical stress. Indeed, the thrombotic risk during laparoscopic surgery is one of the most debated issues and is still quite controversial.

Even though we are not able to state if there will be advantages in choosing a specific pressure setting, we believe that the question is worthy of further studies, considering the fact that the trend observed in the values of some of the parameters is opposite to those reported by other Authors.

If further studies will confirm the results from our study, it will be preferable to use a minor pneumoperitoneum pressure value in order to reduce the thrombotic risk induced by laparoscopy while maintaining an adequate surgical field.
